# Accurate and efficient band gap predictions of metal halide perovskites using the DFT-1/2 method: GW accuracy with DFT expense

**DOI:** 10.1038/s41598-017-14435-4

**Published:** 2017-10-30

**Authors:** Shu Xia Tao, Xi Cao, Peter A. Bobbert

**Affiliations:** Center for Computational Energy Research, Department of Applied Physics, Eindhoven University of Technology, P.O. Box 513, 5600MB, Eindhoven, The Netherlands

## Abstract

The outstanding optoelectronics and photovoltaic properties of metal halide perovskites, including high carrier motilities, low carrier recombination rates, and the tunable spectral absorption range are attributed to the unique electronic properties of these materials. While DFT provides reliable structures and stabilities of perovskites, it performs poorly in electronic structure prediction. The relativistic GW approximation has been demonstrated to be able to capture electronic structure accurately, but at an extremely high computational cost. Here we report efficient and accurate band gap calculations of halide metal perovskites by using the approximate quasiparticle DFT-1/2 method. Using AMX_3_ (A = CH_3_NH_3_, CH_2_NHCH_2_, Cs; M = Pb, Sn, X = I, Br, Cl) as demonstration, the influence of the crystal structure (cubic, tetragonal or orthorhombic), variation of ions (different A, M and X) and relativistic effects on the electronic structure are systematically studied and compared with experimental results. Our results show that the DFT-1/2 method yields accurate band gaps with the precision of the GW method with no more computational cost than standard DFT. This opens the possibility of accurate electronic structure prediction of sophisticated halide perovskite structures and new materials design for lead-free materials.

## Introduction

Hybrid organic-inorganic lead halide perovskites have become the jewel in the crown in the field of photovoltaics since the pioneering work by Kojima *et al*. and Im *et al*.^1,2^. In just four years the power conversion efficiency of solar cells based on hybrid perovskites has rapidly increased from an initial promising value of 9%^3^ to over 22%^4^. In addition to the outstanding performance, simple and cost-effective fabrication, i.e. solution-processing techniques, render them one of the most desirable and most studied semiconductor materials in the field of photovoltaics^5^. Their extraordinary properties, including high carrier mobilities, low carrier recombination rates, and the tunable spectral absorption range are attributed to the unique electronic properties of these materials^5–7^. Thanks to the enormous interest from the scientific community, the field of hybrid perovskites has quickly expanded in terms of types of materials by substituting one or more of the organic or inorganic ions in one of the most studied perovskites, methylammonium lead iodide (CH_3_NH_3_PbI_3_), to obtain the metal halide perovskites AMX_3_: (A = Cs, CH_3_NH_3_, CH_2_NHCH_2_, M = Sn, Pb; X = I, Br, Cl).

Despite the rapid progress made in the last few years in terms of the conversion efficiency, the understanding of the fundamental electronic properties of AMX_3_ perovskites is rather limited. This is especially true for realistic structures in working devices, which often are compounds with mixtures of more than one organic cation, metal cation or halide anion with sophisticated surfaces and interfaces^1–7^. The challenges to fundamental understanding of the electronic structure of AMX_3_ perovskites stem from their rich chemical and physical properties and the interplay of these properties^6,7^. In this context, a first-principles computational approach capable of reliably calculating the materials properties (electronic and thermodynamic) is essential. While standard DFT provides reliable structures and stabilities of perovskites, it severely underestimates the band gaps^8–10^. The relativistic GW approximation has been demonstrated to be able to capture their electronic structure accurately but at an extremely high computational cost.

Several research groups^8–16^ have successfully applied the GW method to predicting electronic structures of AMX_3_ perovskites. Even *et al*.^8^ have identified the importance of a giant spin-orbit coupling (SOC) effect (about 1.0 eV) in the lead iodide perovskites, acting mainly on the conduction band (mainly consisting of Pb states). Taking SOC into account in a relativistic GW approach, Brivio *et al*.^9^ predicted an unconventional band dispersion relation and a Dresselhaus splitting at the band edges in pseudo-cubic CH_3_NH_3_PbI_3_, which indicates a direct-indirect band gap character. This theoretical prediction was recently confirmed^6,7^ by two groups of researchers independently, revealing the mechanism behind the slow charge recombination in these materials. Umari *et al*.^10^ studied the substitution of Pb by Sn and found the SOC effect on Sn perovskites to be much smaller (about 0.4 eV). The same group of researchers also looked at the substitution of halide ions in CH_3_NH_3_PbX_3_^11^ and nicely reproduced experimental findings in optical behavior, such as an increase of the band gap when moving from I to Cl. A few other theoretical studies at GW level include the investigation of polar phonons^12^, crystal structure effects and phase transitions^13,14^, exciton binding energies^15^, and band gap trends^16^ in AMX_3_. The above mentioned GW studies have been very important in understanding the chemistry and physics of these materials and providing materials design inspirations.

However, the GW calculations of realistic structures of metal halide perovskites remain challenging because of i) spin-orbit coupling effects ii) the complex structures and phase transitions in these materials iii) the necessary compromise between the size of the system studied and the extremely high computational cost. This leads to the quest for an accurate and cost effective theoretical framework for electronic structure calculations of realistic AMX_3_ structures and possibly the A/M/X mixed compounds. Here, we apply a recently developed approximate quasiparticle method, namely the DFT-1/2 method^17,18^, which allows us to accurately model the band gaps of AMX_3_ perovskites with minimal computational cost. The DFT-1/2 calculated electronic properties of AMX_3_ perovskites are compared with those calculated with the GW method and with experimental data. Trends in the interplay of geometrical properties and electronic structure properties are analyzed systematically. Our results indicate that the DFT-1/2 method yields band gaps with a GW precision, but with a computational cost similar to standard DFT, opening the way to the study of sophisticated structures and new materials design.

## Computational methods and structural models

**Figure 1:**
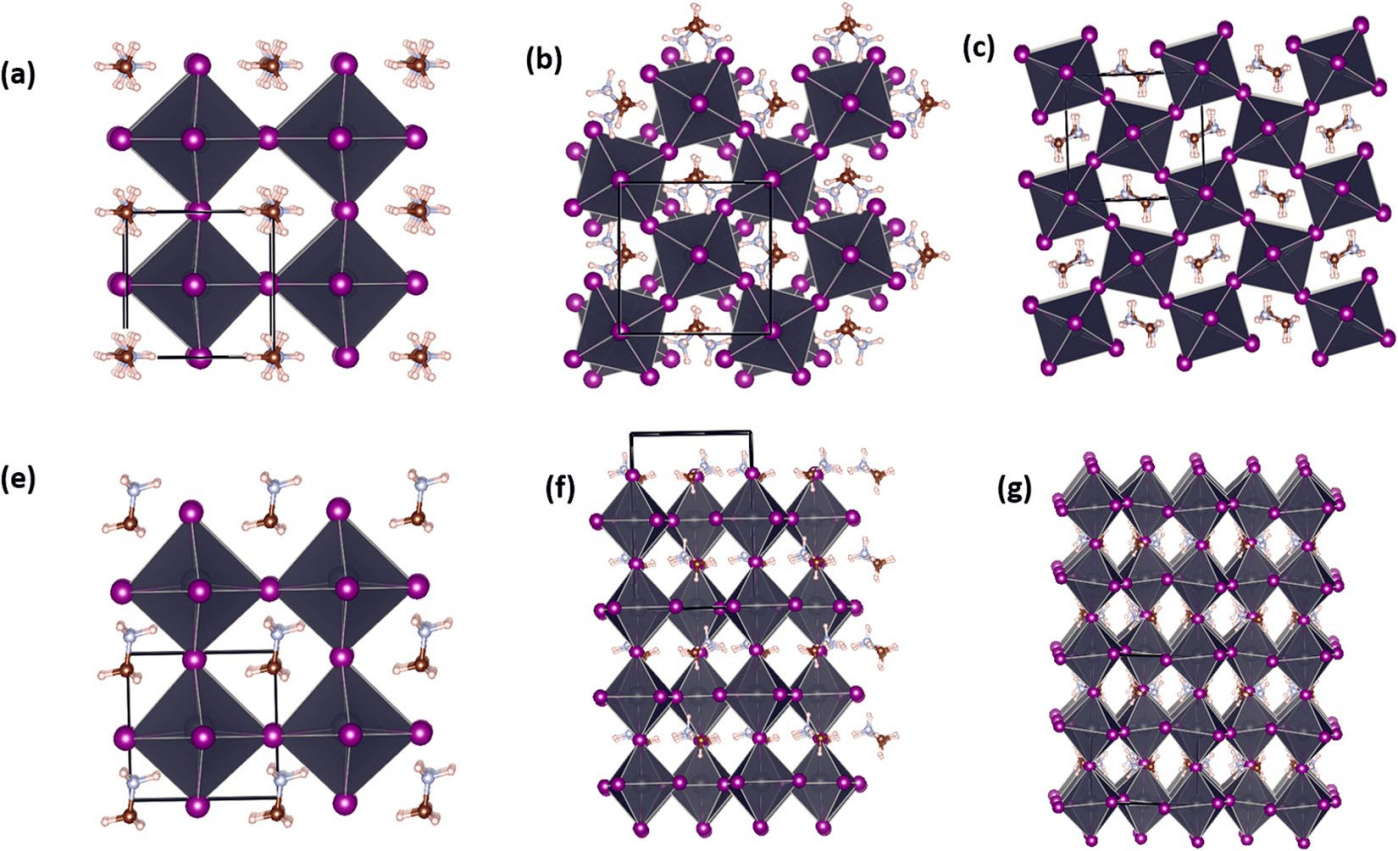
Top (**a**,**b**, and **c**) and side (**e**,**f**, and **g**) views of various structures of metal halide perovskites, taking CH_3_NH_3_PbI_3_ as an example (visualization by VESTA^a^). From left to right: cubic, tetragonal and orthorhombic structures. The unit cell used in the DFT calculations are indicated by black lines ^a^ref.^26^.

The initial structure optimizations are performed using DFT within the local density approximation (LDA)^19^ as implemented in the Vienna *ab-initio* simulation package (VASP)^20^. The exchange-correlation (XC) functional is used as parameterized by Perdew and Zunger^21^. The outermost *s*, *p*, and *d* (in the case of Pb and Sn) electrons are treated as valence electrons whose interactions with the remaining ions is modeled by pseudopotentials generated within the projector-augmented wave (PAW) method^22^. It is well recognized that the orthorhombic↔tetragonal↔cubic (size-dependent, temperature-dependent, composition-dependent) phase transitions in AMX_3_ perovskites can significantly alter their optical as well as electrical properties and thus impact their applications. Therefore, we also considered all three structures. Figure 1 shows the crystal structures and unit cells used in the DFT calculations. Taking CH_3_NH_3_PbI_3_ as an example, unit cells with 12, 48, and (also) 48 atoms are used for the case of cubic, tetragonal, and orthorhombic crystal structures, respectively. During the optimization, the positions of the atoms, and the shape and volume of the unit cell are all allowed to relax. Using cubic CH_3_NH_3_PbI_3_ unit cell as an example, an energy cutoff of 400 eV and 6 × 6 × 6 *k*-point are sufficient for fairly good convergence in total energy and lattice parameters. For the sake of high accuracy, a energy cutoff of 500 eV and 10 × 10 × 10, 8 × 8 × 6, and 6 × 8 × 6 *k*-point meshes (for cubic, tetragonal and orthorhombic structures, respectively) are used to achieve energy and force convergence of 0.1 meV and 2 meV/Å, respectively.

DFT-LDA slightly underestimates the lattice parameters of the AMX_3_ structures by about 1% to 3.5% depending on the composition of the compound and its structure. Usually, such an underestimation of lattice parameters leads to a small overestimation of the band gaps, e.g., by about 70–150 meV for group III/V semiconductors^23^, taking volume deformation potentials into account. However, the opposite is found for metal halide perovskites, namely, an underestimation of the band gaps. The deviations of the band gaps (compared to those with experimental lattice constants) are in the range of 100 meV to 250 meV when using LDA-optimized lattice constants. The inclusion of Van der Waal corrections (DFT-D3 and DFT-TS) resulted in significant improvement of the DFT calculated lattice parameters. However, for each scheme, the degree of improvement are different for different materials. For example, DFT-D3 scheme produce the best results for all inorganic AMX_3_, such as CsSnI_3_ and CsPbI_3_ (slight overestimation of about 0.3%), while DFT-TS performs best results for hybrid AMX_3_, such as, CH_3_NH_3_PbI_3_ (slight underestimation of about 0.1%). Due to a wide range of materials (both hybrid and all inorganic metal halide) included in this work, to be consistent, all the electronic structure calculations were performed with corrected lattice parameters by expanding the lattice parameters proportionally (to match experimental volume of the cells) while keeping the LDA-optimized shape of the cells. This procedure keeps the *ab-initio* aspects of our approach without compromising accuracy. As an example, a band gap difference of 0.04 eV was found for CH_3_NH_3_PbI_3_ when using this procedure (DFT-1/2 band gaps of 1.84 eV *vs* 1.88 eV).

The subsequent electronic structure calculations were performed using the DFT-1/2 method. The DFT-1/2 method stems from Slater’s proposal of an approximation for the excitation energy, a transition state method^24,25^, to reduce the band gap inaccuracy by introducing a half-electron/half-hole occupation. Ferreira *et al*.^17^ extended the method to modern DFT and particularly to solid-state systems, by assuming that the excited electron in the conduction band of a semiconductor usually occupies Bloch-like states with nearly vanishing self-energy, while the hole left in the valence band is localized with a finite self-energy. The self-energy of the hole was corrected by modifying the corresponding pseudopotentials of the atoms (in real space) by removing half an electron from the orbitals that contribute to the top of the valence band^17,18^. The DFT-1/2 method has been demonstrated to be a successful approximate quasiparticle method for a large range of materials, including group III/V semiconductors, intermediate and large band gap nitrides, oxides, and other materials^17,18^. Fortunately, the computational effort is the same as for standard DFT, with a straightforward inclusion of SOC when coupled with VASP^20^.

In this paper, we have applied the DFT-1/2 method (the LDA-1/2 version of ref.^17^) by using an occupation number of eight and proper cutoff radii (CUT) for the M cations and X anions in the AMX_3_ compounds (Table 1). When determining these parameters, we have followed three rules: (i) the parameter CUT is chosen so as to maximize the band gap, ii) this parameter is transferable to different chemical environments in the family of metal halide perovskites, iii) the corrections of the A cations were not included mainly because A cations were demonstrated to not influence the band gap and band edges of metal halide perovskites. A schematic illustration of the application procedure of the DFT-1/2 method included in Fig. 1S in the Supplementary Material. Also shown in Table 1, the location of the valence band maximum are mostly located in the real space around the anions (Cl, Br and I) in a certain radius. The values of the CUT radius show an interesting trend: the larger the halogens ions are, the larger the CUT. To validate the application of the DFT-1/2 method, a direct comparison of the band gaps obtained using the DFT-1/2 method with GW band gaps calculated by Brivio *et al*.^9^ (CH_3_NH_3_PbI_3_, CH_2_NHCH_2_SnI_3_) and Mosconi *et al*.^12^ (AMX_3_: A = CH_3_NH_3_/CH_2_NHCH_2_, M = Pb/Sn, X = I, Br, Cl) are shown in Table 1S of the Supplementary Material. The agreement between the DFT-1/2 and GW band gaps is excellent, with a maximum difference of less than 0.2 eV.

**Table 1:** Values of the parameter CUT (in atomic units) for the half ionized orbitals used within the DFT-1/2 electronic structure calculations.

Atom	CUT (a.u.)	Half-ionized orbital
Pb	2.18	d
Sn	2.30	p
Cl	3.12	p
Br	3.34	p
I	3.76	p

## Results and Discussion

### Comparison of DFT and DFT-1/2 applied to pseudo-cubic CH_3_NH_3_PbI_3_

**Figure 2:**
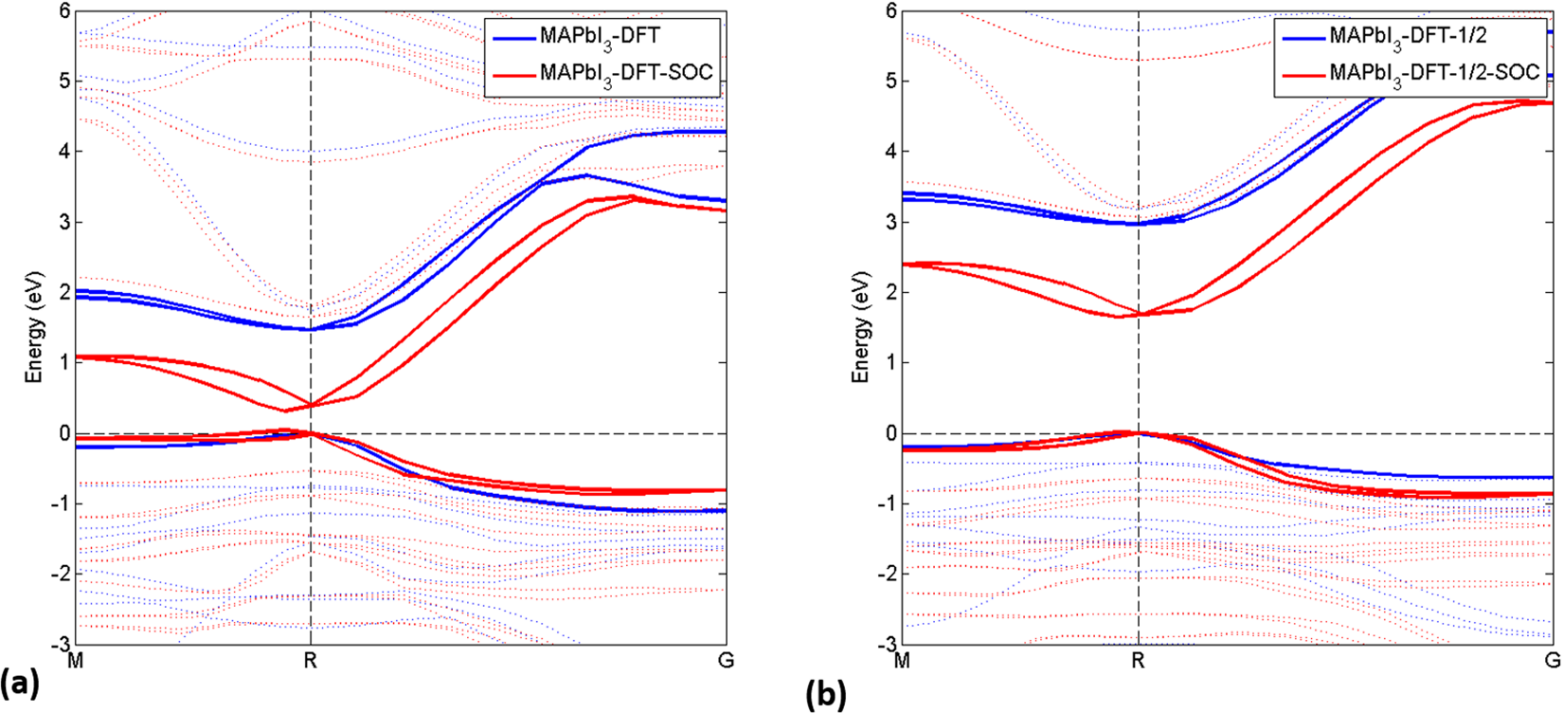
Comparison between calculated band structure of CH_3_NH_3_PbI_3_ with (**a**) DFT (LDA) and (**b**) DFT-1/2 (LDA-1/2) for a pseudo-cubic structure with and without SOC. The highest-lying valence and lowest-lying conduction bands are highlighted as thick solid lines. The energy zero is set in both cases at the highest occupied state. DFT-LDA-optimized lattice parameters (8.542, 8.555, and 12.596) were used.

To compare the performance of DFT-1/2 with standard DFT in electronic structure calculations, we choose as an example the most studied metal halide perovskite CH_3_NH_3_PbI_3_ with a pseudo-cubic structure. It is shown in Fig. 2(a) that DFT (LDA) indeed underestimates the band gap severely, which is found to be 1.47 eV and 0.39 eV without and with SOC, respectively. The tendency of DFT to underestimate band gaps is traditionally associated with the energy cost to excite electron-hole pair. Thanks to the self-energy correction, DFT-1/2 (LDA-1/2) performs extremely well with band gaps of 2.77 eV and 1.68 eV without and with SOC, respectively; see Fig. 2(b). The latter value is only 0.01 eV larger than the value of 1.67 eV calculated by Brivio *et al*.^9^ using the GW approach. Both values are slightly higher than the experimental value of about 1.60 eV^27,28^.

As compared to DFT, both the valence band (VB) and conduction band (CB) calculated with DFT-1/2 shifts downwards and upwards in energy, resulting in a band gap widening of about 1.30 eV both with and without SOC. (see Supplementary Material) In addition, the inclusion of SOC also leads to an indirect gap of 1.64 eV between the highest VB state and lowest CB state, which is 0.04 eV lower than the direct band gap. In comparison to other theoretical predictions of 20 meV of this difference using the HSE06 hybrid fuctional^29^, and 75 meV^7^ using self-consistent GW, our value is the close to the very recent experimental finding of an activation energy of 47 meV^6^. It should be noted that the direct-indirect character of the band gap in CH_3_NH_3_PbI_3_ is also found by us in CH_3_NH_3_PbBr_3_ and CH_3_NH_3_PbCl_3_ for a pseudo-cubic structure, with energy differences of about 0.03 eV (not shown).

### DFT-1/2 results for AMX_3_ (A = CH_3_NH_3_, CH_2_NHCH_2_, Cs, M = Pb, Sn, X = I, Br, Cl)

**Figure 3:**
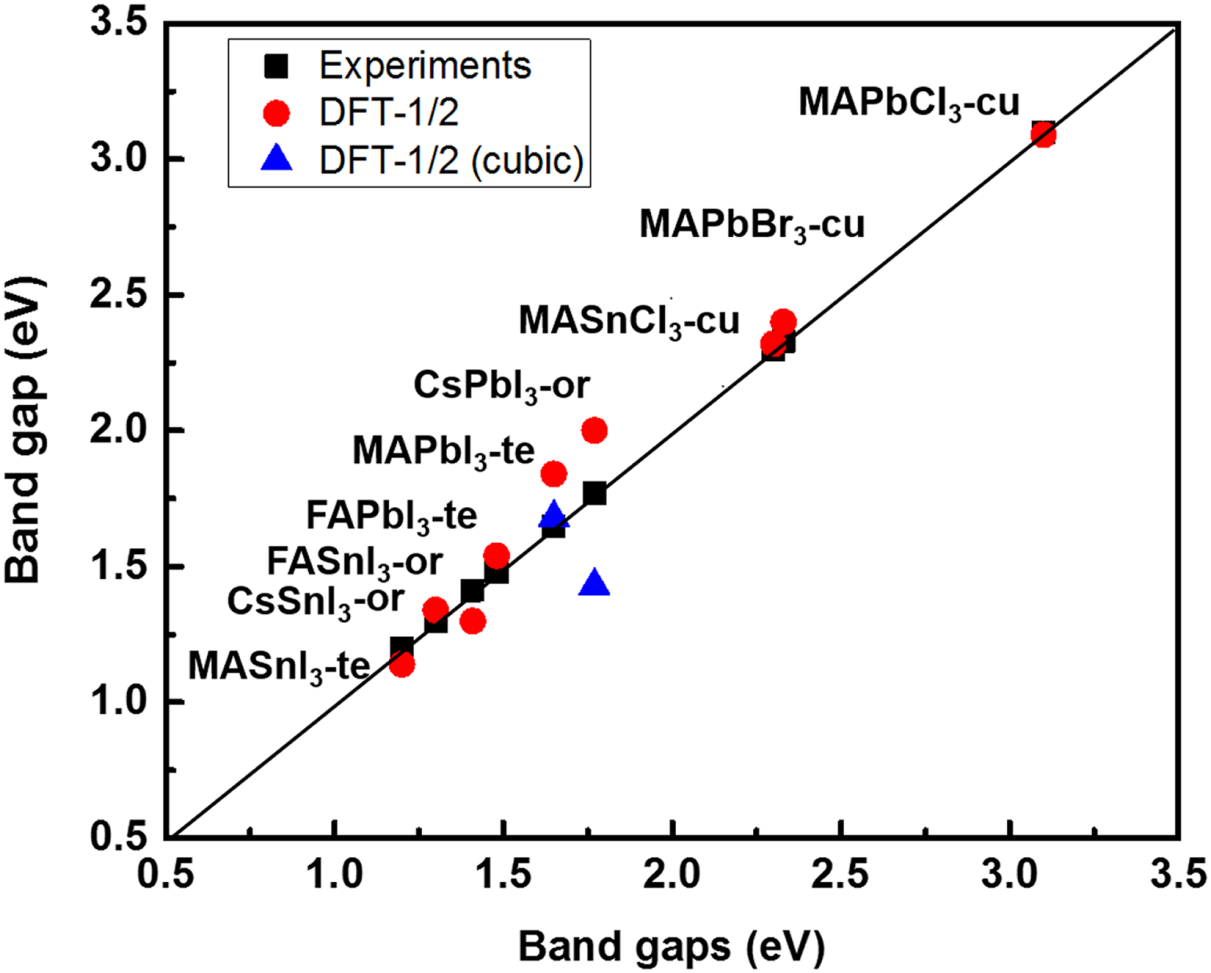
Comparison between experimental and DFT-1/2 band gaps of AMX_3_ perovskites. Note: when the room-temperature crystal structure is uncertain (CH_3_NH_3_PbI_3_ and CSPbI_3_), the band gaps of the high-temperature crystal structure, i.e. the cubic phase, are also plotted. When no experimental result is available (CH_3_NH_3_SnCl_3_), the GW result is used. MA = CH_3_NH_3;_ FA = CH_2_NHCH_2_.

We applied the DFT-1/2 methodto calculate the electronic structure of nine AMX_3_ perovskites with their preferred crystal structures at room temperature^30–36^: i) (pseudo-) cubic for CH_3_NH_3_PbBr_3_^30^, CH_3_NH_3_PbCl_3_^30^, and CH_3_NH_3_SnCl_3_^36^ ii) tetragonal for CH_3_NH_3_PbI_3_^30^, CH_2_NHCH_2_PbI_3_^31^, and CH_3_NH_3_SnI_3_^35^ iii) orthorhombic for CsPbI_3_^32^, CH_2_NHCH_2_SnI_3_^33^, and CsSnI_3_^34^. Table 2 and Fig. 3 summarize DFT-optimized lattice parameters and DFT-1/2 calculated band gaps and their comparison with experimental data and GW results from literature^37–44^.

**Table 2:** Summary of experimental and DFT optimized lattice constants (in Å), band gap energies (eV) obtained with the DFT-1/2 method with and without SOC, compared with relativistic GW and experimental results.

materials	optimized lattices constants^a^	experimental lattice constants	DFT-1/2	DFT-1/2 + SOC	Expt.	GW + SOC
α- CH_3_NH_3_PbI_3_	6.172, 6.149, 6.218 (2.5%)	6.328^d^	2.96	1.81	1.60–1.61,^A–C^, 1.65 ^D^, 1.68^C^	1.31–1.73 ^M,N,O,P,Q^
β- CH_3_NH_3_PbI_3_	8.542, 8.555, 12.596 (2.5%)	8.86, 8.86, 12.66^d^	2.84	1.84		
α- CH_3_NH_3_PbBr_3_	5.539, 5.505, 5.644 (2%)	5.675^d^	3.49	2.40	2.33–2.35^D,E^	2.34^O^, 2.56^Q^, 2.83^R^
α- CH_3_NH_3_PbCl_3_	5.822, 5.802, 5.869 (1.2%)	5.901^d^	4.16	3.09	2.88–3.13^D–G^	3.07^O^, 3.46^Q^, 3.59^R^
α- CH_2_NHCH_2_PbI_3_	6.321, 6.149, 6.216 (2%)	6.362^e^	2.47	1.38	—	—
β- CH_2_NHCH_2_PbI_3_	8.843, 8.843, 12.413 (2%)	—	2.77	1.54	1.41–1.47^H, I^	1.38^M^1.48^O^
α-CsPbI_3_	6.135 (2%)	6.289^f^	2.54	1.44	1.74^J^	1.62^R^
γ-CsPbI_3_	8.959, 12.222, 7.933 (2%)	—	3.05	2.00		—
α- CH_2_NHCH_2_SnI_3_	6.220, 6.053, 6.133 (3.5%)	—	1.40	1.10	—	—
γ- CH_2_NHCH_2_SnI_3_	8.566, 12.097, 8.716 (3.5%) ^b^	8.930, 6.309, 9.062^g^	1.54	1.23	1.41^K^	1.27^O^
α-CsSnI_3_	6.033 (3%)	6.219^h^	1.25	0.82	—	0.60^S^, 1.01^T^
γ-CsSnI_3_	8.649, 12.073, 8.190 (3%)	8.688, 12.378, 8.643^h^	1.71	1.34	1.30^L^	1.30^P^
α- CH_3_NH_3_SnI_3_	6.073, 6.063, 6.133 (2.5%)	6.230, 6.230, 6.232^i^	1.29	0.91	—	1.03^O^, 0.89^R^
β- CH_3_NH_3_SnI_3_	8.432, 8.442, 12.377 (2.5%)	8.758, 8.758, 12.429^i^	1.52	1.14	1.21^U^	1.10^N^
α- CH_3_NH_3_SnCl_3_	5.496, 5.401, 5.564 (2.5%)	5.760^j^	2.63	2.32	—	2.30^R^
γ - CH_3_NH_3_SnCl_3_	7.236, 10.961, 8.098 (2.5%)^c^	7.910, 5.726, 8.227^j^	2.53	2.22	—	—

^d^ref.^30^, ^e^ref.^31^, ^f^ref.^32^, ^g^ref.^33^, ^h^ref.^34^, ^i^ref.^35^, ^j^ref.^36^, ^A^ref.^27^, ^B^ref.^28^, ^C^ref.^35^, ^D^ref.^36^, ^E^ref.^45^, ^F^ref.^37^, ^G^ref.^38^, ^H^ref.^39^, ^I^ref.^40^, ^J^ref.^41^, ^K^ref.^42^, ^L^ref.^43^, ^M^ref.^9^, ^N^ref.^10^, ^O^ref.^12^, ^P^ref.^14^, ^Q^ref.^11^, ^R^ref.^16^, ^S^ref.^13^, ^T^ref.^15^, ^U^ref.^44^. ^a^Values in brackets are the expansion percentages used to match the DFT-optimized unit cell volumes to the experimental ones. ^b,c^Experimentally the triclinic structure is observed at room temperature. However, to be consistent with other systems, the orthorhombic unit cell was used in the DFT calculations (the lattice constant *b* is doubled). α, β, and γ denote, respectively, (pseudo-) cubic, tetragonal and orthorhombic structures of AMX_3_. The details of the DFT-optimized crystal structures are given in the Supplementary Material.

Despite the approximation made in the procedure of adapting to the experimental lattice parameters (see Section computational methods) and the use of relatively small unit cells of the structures, the DFT-1/2 band gaps are in good agreement with experimental and GW results. The maximum discrepancy is about 0.2 eV, with the majority of the band gaps slightly overestimated and a few slightly underestimated. Special attention needs to be paid to the case of CsPbI_3_ due to the fact that the room-temperature crystal structure is uncertain, which is an indication of a metastable phase at the experimental conditions. Therefore, one has to be careful when comparing a band gap value obtained from theoretical calculations for a certain structure that is different in experiments. The relatively large overestimation of the band gap of CsPbI_3_ can be understood by assuming in the experiment an intermediate configuration between a cubic and orthorhombic assembling structure. Indeed, the average band gap of cubic (α-) CsPbI_3_ and orthorhombic (γ-) CsPbI_3_ is 1.72 eV, which is only 0.02 eV lower than the experimental value of 1.74 eV.

A few general trends are observed from Fig. 3 and Table 2:

i) For the same composition, the band gap decreases with increasing ordering in the crystal, namely orthorhombic >tetragonal >cubic. The magnitude of the differences is generally small for systems with CH_3_NH_3_, intermediate for CH_2_NHCH_2_, and large for Cs cations. Two extreme cases are CH_3_NH_3_PbI_3_ with a difference of 0.03 eV (cubic *vs* tetragonal) and CsSnI_3_ with a difference of 0.52 eV (cubic *vs* orthorhombic). The small band gap value of CsSnI_3_ in a cubic structure was also predicted using the GW method^13,15^ (DFT-1/2: 0.82 eV, GW: 0.60 eV and 1.01 eV). This is probably related to the strong relaxation and reorientation of the organic cation (CH_3_NH_3_, CH_2_NHCH_2_) within the inorganic framework, resulting in pseudo-cubic structure (which is very close to a tetragonal structure), whereas the effect of Cs on the crystal structure is small, maintaining the perfect cubic structure.
ii) In general, the band gap increases with an increase of electronegativity of the M and X ions: from I to Br to Cl and from Pb to Sn.
iii) There is no general trend when considering the effect of the A cations. The effect of the A cations will be discussed in the following band structure analysis.

### Electronic band structures and Density of States (DOS)

**Figure 4:**
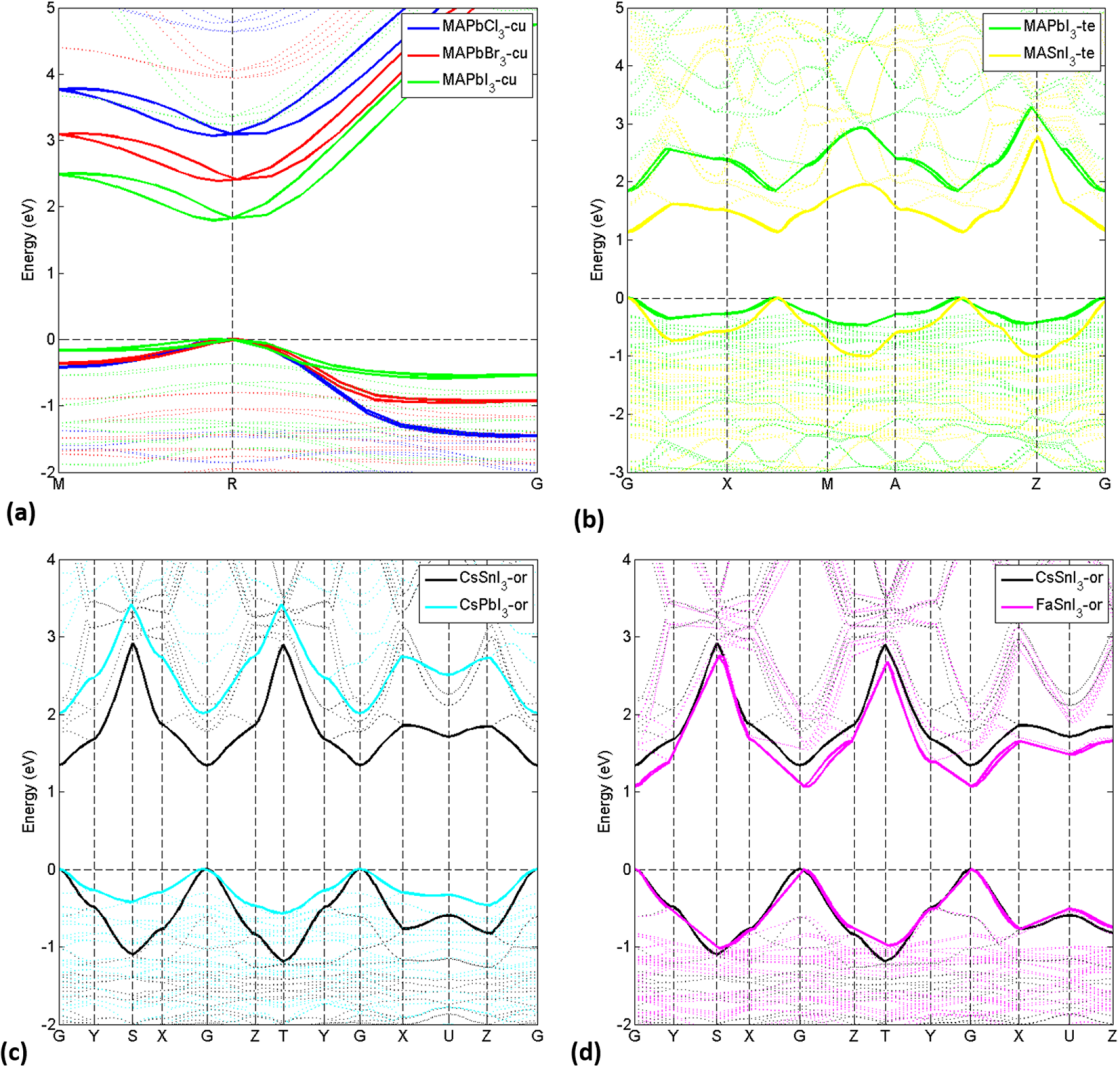
Calculated DFT-1/2 band structure (VBM and CBM highlighted as thick solid lines) for (**a**) CH_3_NH_3_PbX_3_ (X = I, Br, Cl) in a pseudo-cubic structure, (**b**) CH_3_NH_3_MI_3_ (M = Sn, Pb) with a tetragonal structure, (**c**) CsMI_3_ (M = Sn, Pb) with an orthorhombic structure, (**d**) ASnI_3_ (A = Cs, CH_2_NHCH_2_) with an orthorhombic structure. The energy zero is set in both cases at the highest occupied state. MA = CH_3_NH_3;_ FA = CH_2_NHCH_2_.

To provide a better insight into the trends in the electronic structures of the nine AMX_3_ perovskites, we plot the electronic band structures in Fig. 4. We group the compounds in terms of crystal structures and variations in only one of the ions (A/M/X): CH_3_NH_3_PbX_3_ (X = I, Br, Cl) with a pseudo-cubic structure, CH_3_NH_3_MI_3_ (M = Pb, Sn) in a tetragonal structure, CsMI_3_ (M = Sn, Pb) with an orthorhombic structure, ASnI3 (A = Cs, CH_2_NHCH_2_) with an orthorhombic structure.

For CH_3_NH_3_PbX_3_ (X = I, Br, Cl) with a pseudo-cubic structure, see Fig. 4(a), the band gaps and the trend in the increasing band gap when moving from I to Cl are in excellent agreement with experiments. The maximum discrepancy is less than 0.02 eV, which is within the inherent accuracy of the methodology. The agreement of our results with experiments, especially for the cases of CH_3_NH_3_PbBr_3_ and CH_3_NH_3_PbCl_3_, are better than in the work of Mosconi *et al*.^11^ using the GW method, where sizable overestimations were found for a tetragonal structure. This again highlights the importance of the crystal structure of the studied materials when comparing with experiments. The common feature of all the simulated band structure are significant SOC effect with direct-indirect band gaps. The substitution of I by Br (Cl) has little influence on the band dispersion of the CB (consisting mainly of Pb 6p states) but leads to sizable changes in the VB (consisting mainly of halide *n*p states and to some extent of Pb 6 s states). More dispersion in the VB is found when substituting I by Br (Cl), evidenced by the downshifting of the bands at the Γ point (Fig. 4(a)).

**Figure 5:**
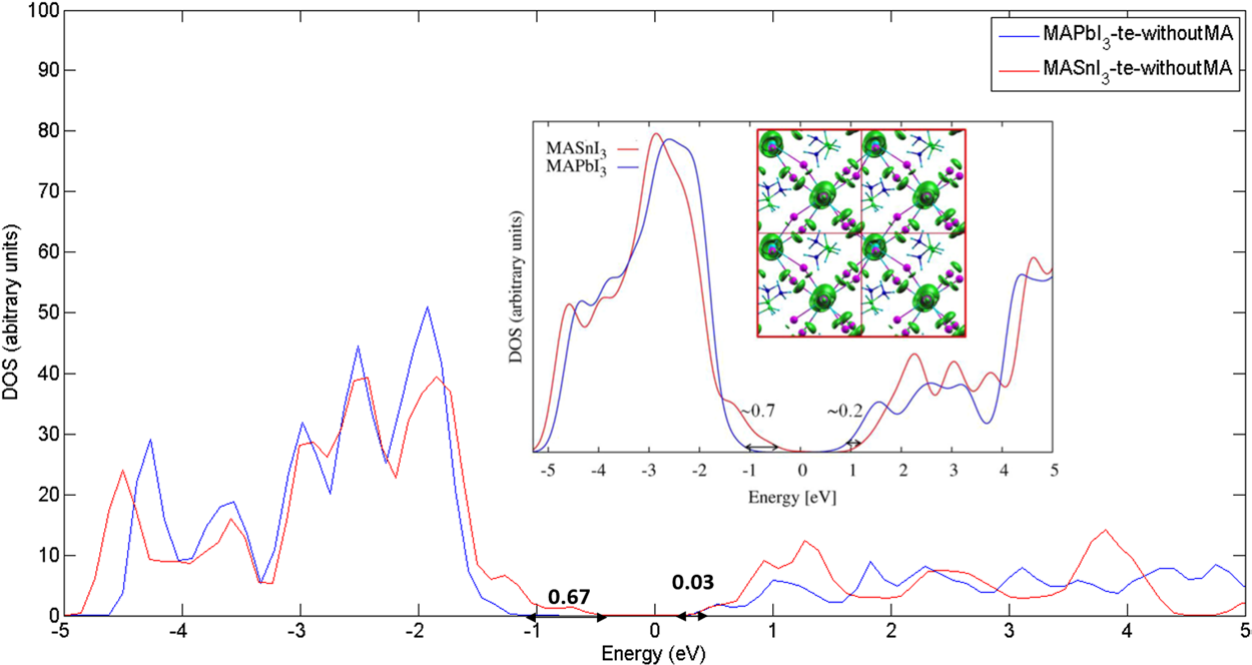
Electronic DOS for CH_3_NH_3_PbI_3_ (blue) and CH_3_NH_3_SnI_3_ (red) calculated by DFT-1/2. The DOS peaks have been aligned at the localized I states at about -13 eV (Fig. 3S in the Supplementary Material). Inset: SOC-GW calculated DOS of CH_3_NH_3_PbI_3_ and CH_3_NH_3_SnI_3_ from Umari *et al*.^10^. **MA** = **CH**_**3**_ **NH**_**3**_ **;** **FA** **=** **CH**_**2**_ **NHCH**_**2**_.

We pay special attention to two very important materials for photovoltaic applications, tetragonal CH_3_NH_3_PbI_3_ and CH_3_NH_3_SnI_3_. Figures 4(b) and 5 show the band structures and densities of states (DOS), respectively, together with a comparison with results from Umari^10^. As shown in Table 2, the band gap of 1.14 eV of CH_3_NH_3_SnI_3_ is in excellent agreement with the experimental value of 1.21 eV and the GW value of 1.10 eV. Similar to the case of CH_3_NH_3_PbX_3_, the CB dispersion characteristics are almost unchanged. The substitution of Pb by Sn leads to a slight shift of the CB to higher energy by about 0.03 eV (see Fig. 5). On the contrary, the VB maximum is shifted upwards much more strongly by about 0.67 eV and the band dispersion increases (see the band dispersion between Γ and X, M and A, and at Z). These electronic structure differences between CH_3_NH_3_PbI_3_ and CH_3_NH_3_SnI_3_ agree nicely with experimental findings in optical absorption spectra of a red-shift and an increased absorption intensity near the absorption onset^42^. In addition, our calculated DOS and band edge alignment is in good agreement with results using the GW method by Umari *et al*.^10^: 0.67 (0.03) *vs* 0.7 (0.2) eV for VB (CB) energy shifts.

For CsMI_3_ (M = Sn, Pb) with an orthorhombic structure (Fig. 4(c)), a very similar trend is found as for CH_3_NH_3_MI_3_ (M = Sn, Pb). The substitution of Pd by Sn leads to a smaller band gap. It should be noted that the DFT-1/2 band gaps of CsMI_3_ are slightly larger than those of CH_3_NH_3_MI_3_: 2.00 (1.30) eV *vs* 1.81 (1.14) eV for M = Pb (Sn). The difference in band gaps between CsMI_3_ and CH_3_NH_3_MI_3_ could be a result of variation in structures, unit cell sizes, and the detailed interactions of the A, M, and I ions. Common explanations for the difference are counteracting each other. For example, the volume of the CsPbI_3_ unit cell is 7% smaller than that of the unit cell of CH_3_NH_3_PbI_3_ (usually leading to a smaller band gap), whereas the degree of the tilting of the PbI_6_ octahedral framework in CsPbI_3_ is larger than that for CH_3_NH_3_PbI_3_ (usually leading to a larger band gap). While both the volume and the tilting of the octahedral framework of CsSnI_3_ are slightly smaller than those of the MASnI_3_by less than 2% (both usually leading to a smaller band gap), the calculated band gap of CsSnI_3_ is in fact larger than that of CH_3_NH_3_SnI_3_. This unexpected result may be explained by taking into account the different chemistry and interactions of Cs and CH_3_NH_3_ with the inorganic matrix of AMX_3_.

As expected, for ASnI_3_ (A = Cs, CH_2_NHCH_2_) with an orthorhombic structure (Fig. 4(d)), the change of cation from Cs to CH_2_NHCH_2_ yields almost no change in band dispersion and a slight band gap decrease. This is in agreement with one of the commonly accepted features of metal halide perovskites: the A cation only weakly interacts with the inorganic matrix, with little electronic contribution at the band edges, and mainly plays a role in changing the volume of the lattice. The band gap differences between CH_2_NHCH_2_SnI_3_ and CH_3_NH_3_SnI_3_ are again due to combinations of differences in unit cell volume (CH_2_NHCH_2_SnI_3_ > CsSnI_3_), tilting of the octahedral framework (CH_2_NHCH_2_SnI_3_ > CsSnI_3_), and chemical bonding characteristics. To summarize, our analysis emphasizes the fact that the electronic structure of AMX_3_ perovskites is a result of the interplay of several properties: chemical environment, crystal structure, unit cell size, and thermodynamics of the materials. Therefore, an accurate electronic structure description of AMX_3_ perovskites requires not only a reliable *ab-initio* method but also a proper understanding of the interplay of several chemical and physical properties of the materials.

In conclusion, we have applied an approximate quasiparticle method, the DFT-1/2 method, to electronic structure calculations of AMX_3_ perovskites (A = CH_3_NH_3_, CH_2_NHCH_2_, Cs; M = Pb, Sn, X = I, Br, Cl) with several crystal structures. Our results show that the DFT-1/2 method yields accurate band gaps showing very good agreement with experimental findings and results from the computationally much more demanding GW method. Nevertheless, the computational cost is comparable to that of a standard DFT method. In addition, the trends in electronic structure properties were identified by varying only one of the three A/M/X ions. In general, the band gaps increase with an increase in electronegativity of the M and X ion, and increase with a decrease of the symmetry of the crystal structures: cubic < tetragonal < orthorhombic. There is no single applicable rule governing the trend in band gaps when only varying the A cation. Here, the interplay of several aspects, such as unit cell size, crystal structure, chemical bonding, and thermodynamics of the material is decisive. Our work demonstrates the success of the DFT-1/2 method in predicting the electronic structure of the AMX_3_ perovskites with minimal computational cost and opens the pathway towards studying large and complex structures in working devices and efficiently designing new metal halide perovskite materials for solar cell applications.

## Electronic supplementary material

Supplementary Information
