# Supplementary material for: Accurate and efficient band gap predictions of metal halide perovskites using the DFT-1/2 method: GW accuracy with DFT expense
Source: Sci Rep. 2017 Oct 30;7:14386. doi: 10.1038/s41598-017-14435-4 (PMC5662598; doi:10.1038/s41598-017-14435-4)
Supplement: Supplementary file 1 — Supplementary Information [file 41598_2017_14435_MOESM1_ESM.pdf]

Supplementary information to "Accurate and efficient band gap predictions of metal halide perovskites using the DFT-1/2 method: GW accuracy with DFT expense"

S. X. Tao[1*], X. Cao[1], P. A. Bobbert[1]

Table 1. Direct comparison of band gaps obtained using DFT-1/2 method with GW results from Brivio et al[1] ($MAPbI_3$, $FASnI_3$) and from Mosconi et al [2] (AMX_3: A=MA/FA, M=Pb/Sn, X=I, Br, Cl). Note the crystals structures of GW work were used directly for DFT-1/2 band gap calculations.

	DFT-1/2 *vs* GW Ref 1	DFT-1/2 *vs* GW Ref 2
$MASnI_3$		1.07 (1.03)
$MAPbI_3$	1.62(1.67)	1.74 (1.67)
$FAPbI_3$		1.66 (1.48)
$MAPbBr_3$		2.40 (2.34)
$MAPbCl_3$		3.10 (3.07)
$FaSnI_3$		1.21(1.27)

1. Brivio, F., Butler, K. T., Walsh, A., & Van Schilfgaarde, M. Relativistic quasiparticle self-consistent electronic structure of hybrid halide perovskite photovoltaic absorbers. Physical Review B 89, 155204, doi:10.1103/PhysRevB.89.155204 (2014).

2. Bokdam, M., Sander, T., Stroppa, A., Picozzi, S., Sarma, D. D., Franchini, C., & Kresse, G. Role of Polar Phonons in the Photo Excited State of Metal Halide Perovskites. Scientific Reports 6, doi: 10.1038/srep28618 (2016).

Figure 1. A schematic illustration of the procedure of the DFT-1/2 method in predicting band structure of the pseudo-cubic $MAPbI_3$ perovskites.

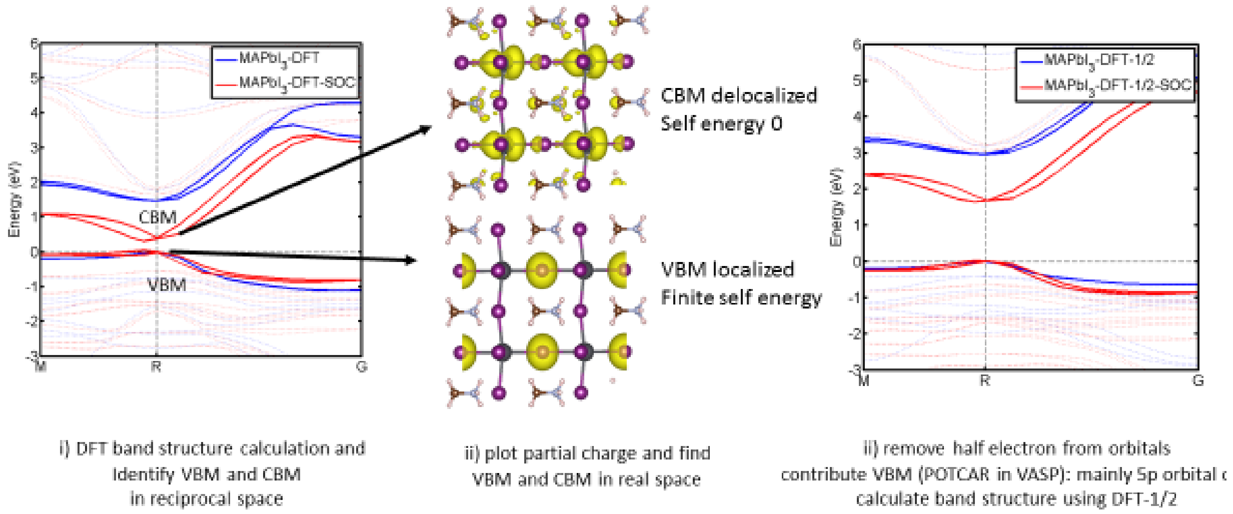

Figure 2 Band structure comparison of $MAPbI_3$ using DFT and DFT-1/2 without aligning the Valence band maximum to 0.

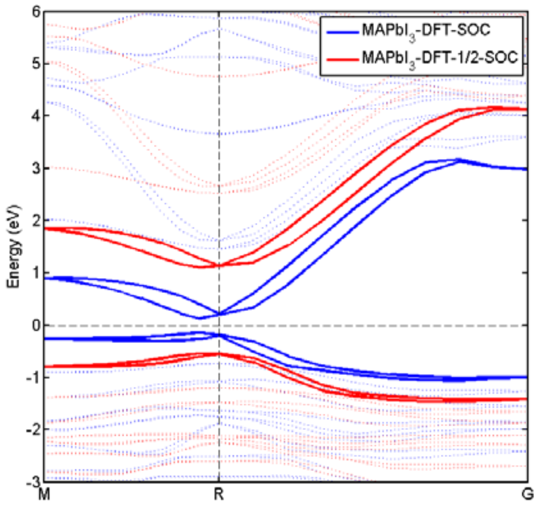

Figure 3 Electronic DOS for $MAPbI_3$ (blue) and $MASnI_3$ (red) calculated by DFT-1/2 (energy range of -25 eV to 10 eV). The DOS peaks have been aligned at the localized I states at about -13 eV.

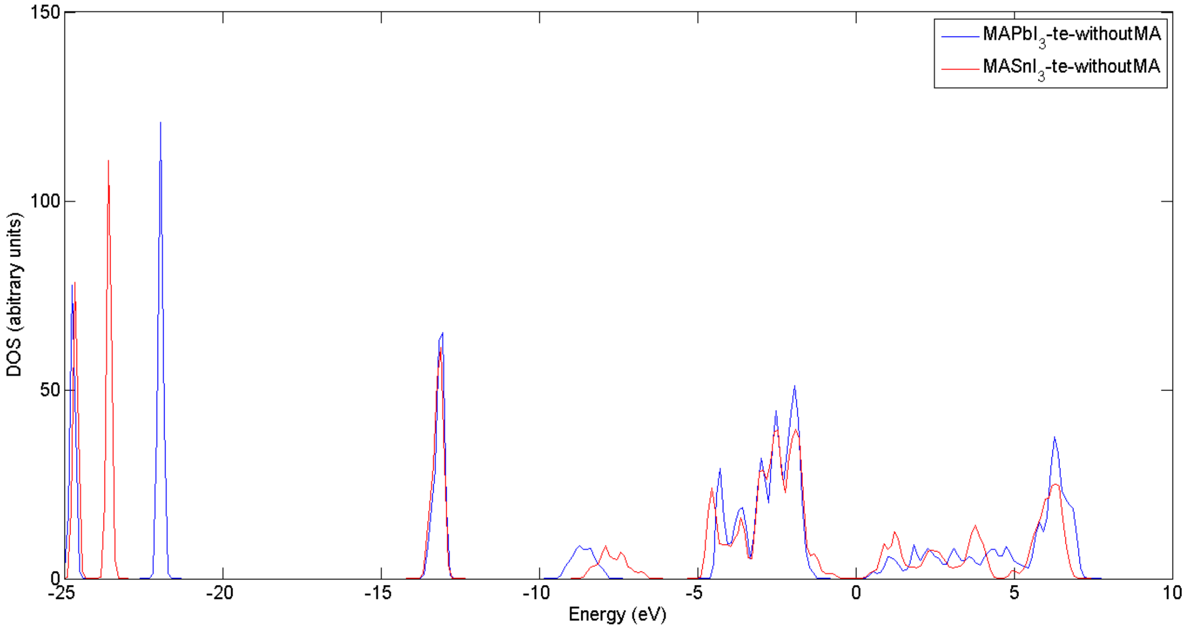

Optimized crystal structures formatted as VASP input file used in DFT-1/2 band structure calculations list in Table 2 in the manuscript:

```
MAPbI3-cubic
   1.02500000000000
     6.1717394338931628   -0.0008531882374086   -0.0501585885085331
    -0.0008500076402037    6.1395863452849246   -0.0017851626838633
    -0.0503636493782260   -0.0017745042043623    6.2184346873496246
   C    N    H    Pb   I
     1     1     6     1     3
Direct
  0.9007168513900297  0.9999345777638595  0.9910526733883387
  0.1357170184255878  0.9996953788852068  0.0343372829410100
  0.8688585974588960  0.9996782355626976  0.8162393690239895
  0.8298756584273477  0.1470403438399472  0.0632866594003190
  0.8296216200505384  0.8531616513752454  0.0637072890186232
  0.2120804707301787  0.1378552707506628  0.9713724290568067
  0.2118653096297933  0.8613247821438677  0.9717113719033321
  0.1744096796530812  0.9999300129253115  0.1995764280873118
  0.4869999191442815  0.5002704598543701  0.4808893771358029
  0.4382965967767589  0.4999387017890840  0.9775135980187386
  0.4385244634437342  0.0002746974115269  0.5226928142736895
  0.9748428438697800  0.5001668976982074  0.4277616977520395
```

MAPbI3-tetragonal

1.0250000000000000

8.5415512571730385 0.0018830583307183 -0.0071806817532417

0.0085434065676154 8.5551944276888925 -0.0038934495258479

0.1002173780274798 0.0764313213134684 12.5961575046758263

C N H Pb I

4 4 24 4 12

Direct

0.0296868046819654 0.4976872446520417 0.2502455073755883

0.5322070288555878 0.0458578332664885 0.2498911066309191

0.0453407152028618 0.5328029454638070 0.7491792621135360

0.4978644807673902 0.0302492564135761 0.7505469944809917

0.9260752069455549 0.6079861523570500 0.1949687551622645

0.4281071909336873 0.9335109319068167 0.1979147343360950

0.9342638262333480 0.4270875187270917 0.6974564314387806

0.6073524767287566 0.9254538024452330 0.6953367116349796

0.6544776764267013 0.0197735953964298 0.2274330955993307

0.5175286121221561 0.0355047437770750 0.3366653401111392

0.5015625576087288 0.1649673116909796 0.2232611623828262

0.4532970048051865 0.8175859618659089 0.2199526683332209

0.4396342277609193 0.9402473279412575 0.1153810558696406

0.3097226810141294 0.9546846482751121 0.2161429016602128

0.9360572836629544 0.5957553163895071 0.1126626844912835

0.9529121702683838 0.7248348997455167 0.2129068782896013

0.8078069737922533 0.5890969820352439 0.2147485517468297

0.1519218579776904 0.5208364272673549 0.2262680388177856

0.9977344710413121 0.3772173580150593 0.2282052470981242

0.0160455930475578 0.5129198026800168 0.3366542843050269

0.9539627029102959 0.3096536221325792 0.7184552533675941

0.9447657303213006 0.4341958889896702 0.6148805273227183

0.8175031573747376	0.4538946765757359	0.7169639554048430
0.1654364290027388	0.5003770852426257	0.7252548935440402
0.0315997698389694	0.5221628377916758	0.8360107277031972
0.0208442618777624	0.6539100737999064	0.7237487190827636
0.7246804507639126	0.9528024640925992	0.7118119539305496
0.5932355025331972	0.9330000169493289	0.6130736986300036
0.5889493051005630	0.8078446475102155	0.7165659578695482
0.5205548846131904	0.1516714700773463	0.7249874703239740
0.5147040005903492	0.0188179513111564	0.8369278800460833
0.3768113524687777	0.9977223310251375	0.7300765433937784
0.5092991392396584	0.5161104582173337	0.9888240789665872
0.5170377983743322	0.5101708882156970	0.4888165075324551
0.0296757111748249	0.0117638428962650	0.4898746508355032
0.0107165047973155	0.0295990521928147	0.9899093535600372
0.7003676003705834	0.8333652693744611	0.9949361905493603
0.3283229593061634	0.2051690134337534	0.9978877294952753
0.2051423522933149	0.7178159990791428	0.9991227281632789
0.8341064710660575	0.3470987376852506	0.9893416892492723
0.3471068455790700	0.8341797681447645	0.4895693405043957
0.7174528129028630	0.2055979374525450	0.4997599311188239
0.8339474806398144	0.7013754087403044	0.4951825901085911
0.2054387616907150	0.3289275027440368	0.4969014482428307
0.5221077370772633	0.5293229615830555	0.2401512535509411
0.5294315622363115	0.5221232972172203	0.7401440582814232
0.0091920552054177	0.0200349575165646	0.7415109482564759
0.0200060957733754	0.0092277656972470	0.2414863580874496

MAPbBr3-cubic

1.01200000000000

5.9199999999999999 0.0000000000000000 0.0000000000000000

0.0000000000000000 5.9199999999999999 0.0000000000000000

0.0000000000000000 0.0000000000000000 5.9199999999999999

C N H Pb Br

1 1 6 1 3

Direct

0.8962732309267807 0.9999835575561917 0.9885225897978458

0.1421229416578313 0.9998276714532324 0.0326818543098426

0.8669576230289806 0.9997673821304502 0.8051964671801670

0.8224554777663400 0.1524554610094526 0.0646689097381810

0.8222031293734631 0.8478330826230547 0.0650649063082653

0.2212941063647946 0.1424140526496274 0.9656588034540547

0.2210818272207788 0.8569803126825306 0.9659456519655976

0.1804492545013900 0.9999679128432390 0.2053791894684025

0.4753317996463835 0.5000043839523016 0.4779234319137089

0.4287771039399573 0.4998766652154316 0.9726919535286029

0.4334819150903257 0.0000195562303063 0.5128482885866177

0.9675529784829351 0.5001724086541017 0.4392056937486757

MAPbCl3-cubic

1.02000000000000

5.5391045274592825 0.0003468956896641 -0.0010448343194384

0.0003448753182629 5.5052137797524301 0.0001664298461292

-0.0013785306998623 0.0001752310013020 5.6439202901432122

C N H Pb Cl

1 1 6 1 3

Direct

0.8899586145506504 0.9999611127432928 0.9746996321370602

0.1446557781237630 0.9998328693923924 0.0449578418890582

0.8771793266459511 0.9998495446299316 0.7798749649489167

0.8020933139467843 0.1645140612286227 0.0452580169852510

0.8019401384725882 0.8356367022449902 0.0454669481036447

0.2358990065205688 0.1540137764715652 0.9823668497221618

0.2357756327953524 0.8455675023478406 0.9825229143105929

0.1692202440177226 0.9999137203198316 0.2298624101125810

0.4820195458734844 0.4999994259267098 0.4829510655592770

0.4383591198597827 0.4998330362334400 0.9753494202562720

0.4185616216959005 0.9999871121174451 0.5341565224073932

0.9823190454974124 0.5001935833438793 0.4183211535678311

$FAPbI_3$-cubic

1.020000000000000

6.3211144246449447 -0.0000000157951959 0.0000000000000000

0.0000008075629942 6.1488880004277604 0.0000000000000000

0.0000000000000000 0.0000000000000000 6.2156829632934336

C N H Pb I

1 2 5 1 3

Direct

0.5000010130025601 0.5220356819570853 0.5000000000000000

0.6833221224189714 0.4256937661911380 0.5000000000000000

0.3166759105786099 0.4256937661860505 0.5000000000000000

0.5000000000005471 0.7009747178519632 0.5000000000000000

0.8157884239113073 0.5224359538161281 0.5000000000000000

0.7038211913288990 0.2596793368997012 0.5000000000000000

0.2961788086693744 0.2596793368993673 0.5000000000000000

0.1842115760901422 0.5224359538145862 0.5000000000000000

0.9999999999998934 0.9647834082093283 0.0000000000000000

0.4999999999999218 0.9837789138322748 0.0000000000000000

0.9999999999998010 0.4622583035517493 0.0000000000000000

0.9999999999999645 0.9190658117906239 0.5000000000000000

$FAPbI_3$-tetragonal

1.020000000000000

8.8399587465740872 -0.0013088808757011 -0.0000046974807339

-0.0013088639889776 8.8399578084821986 -0.0000001502236795

-0.0000066998903198 -0.0000002309632277 12.4200976915162187

C N H Pb I

4 8 20 4 12

Direct

0.7746435153409925 0.2746449060650629 0.2003670471117680

0.2711261399878776 0.7711248044750924 0.3007342335893508

0.7746430580767620 0.2746450323147297 0.7996317557940844

0.2711257386760722 0.7711223563130301 0.6992659855842422

0.6816357376202636 0.1816363191025259 0.2475647454306364

0.1782194550161230 0.8641466013626504 0.2534499672510961

0.6816349665263020 0.1816364299470777 0.7524342007467655

0.1782188863469763 0.8641443683557698 0.7465503757549949

0.8676578618391081 0.3676584617663631 0.2474699477030123

0.3641477228628140 0.6782180737527591 0.2534498331818164

0.8676575687691330 0.3676587753664792 0.7525292693297934

0.3641475178650473 0.6782159389516810 0.7465502466613771

0.8799445494498562 0.3799443704599036 0.3297223727991794

0.3766992796960714 0.6663797150286365 0.1711603679175100

0.8799443579873951 0.3799442013574919 0.6702768981844190

0.3766999121297549 0.6663778588717880 0.8288396813718298

0.9344810754575056 0.4344818298124393 0.1990459618909138

0.4310582020585884 0.6112024150975233 0.3016504197603562

0.9344810589706463 0.4344816199178362 0.8009536728790765

0.4310591191893166 0.6112011833655606 0.6983496601732597

0.6693680407373164 0.1693678292004197 0.3298118958450473

0.1663806942996040 0.8766984655659876 0.1711604258409424

0.6693673531279465 0.1693677931793632 0.6701871617109356

0.1663811628229934 0.8766965196133091 0.8288397780088764

0.6147228373430703 0.1147236390125636 0.1992684927730775

0.1112037981567041 0.9310574012509422 0.3016504280881107

0.6147225772068775 0.1147232435184856 0.8007304677863140

0.1112044773297577 0.9310559643523985 0.6983497241292345

0.7745300924432077 0.2745320428547652 0.1115531261797778

0.2707173401039470 0.7707161873408765 0.3895207253702290

0.7745299833791898 0.2745319712492315 0.8884459579814887

0.2707173139570478 0.7707142549114633 0.6104791846648372

0.2727911064503869 0.2716471586547899 0.4999999582970716

0.2732369289931407 0.2722687948180227 0.9999999482394357

0.7716477056416657 0.7727906527675341 0.5000004497242211

0.7722691155964032 0.7732367873267253 0.0000004294655155

0.5470691086499139 0.0470677489878385 0.4999995337610246

0.0020175083391995 0.5020159322672415 0.5000001647324872

0.5070719526450486 0.5385138383964089 0.4999999409777253

0.0385168234235788 0.0070689153527740 0.5000003161629971

0.5428653751600229 0.0428648495033139 0.9999998385324955

0.0072381229315927 0.5072374909853751 0.9999996203292063

0.4987143269590430 0.5479050038254458 0.0000004333818766

0.0479069694746347 0.9987124778232942 0.9999997989052872

0.2735032906527326 0.2713816740976678 0.7500031798824776

0.2735035310747365 0.2713833127161021 0.2499965630375837

0.7713698963810640 0.7735073398892371 0.7500045865788579

0.7713828968526043 0.7735033288539945 0.2499981864973736

$CsPbI_3$-orthorhombic

1.02000000000000

8.9599414455581332 0.0000000000000000 -0.0019858272315529

0.0000000000000000 12.2225883518336147 0.0000000000000000

-0.0017217251913990 0.0000000000000000 7.9327952756357982

Cs Pb I

4 4 12

Direct

0.0969151594643876 0.2500000000000000 0.9530078951339789

0.9033814508768998 0.7500000000000000 0.0467686762672770

0.4033051129230714 0.7500000000000000 0.4532687677607186

0.5970795436661973 0.2500000000000000 0.5468552783607521

0.5000457781896586 0.4999462016998493 0.0000371185473611

0.0001531021329768 0.4999511693573453 0.5001021746821408

0.5000457781896586 0.0000537983001507 0.0000371185473611

0.0001531021329768 0.0000488306426547 0.5001021746821408

0.1863979457335461 0.5399207631884479 0.1694683722032480

0.8137584223543968 0.4600197733542970 0.8304861121995089

0.3137272263034205 0.4602277904076715 0.6694774311414946

0.6865060990948422 0.5396946570264802 0.3304969521010790

0.8137584223543968 0.0399802266457030 0.8304861121995089

0.1863979457335461 0.9600792368115521 0.1694683722032480

0.6865060990948422 0.9603053429735198 0.3304969521010790

0.3137272263034205 0.0397722095923285 0.6694774311414946

0.4960645257050089 0.2500000000000000 0.0835340882284186

0.5040218884636261 0.7500000000000000 0.9168994709025000

0.0036421718080888 0.7500000000000000 0.5829772433238887

0.9963673345995687 0.2500000000000000 0.4165522532728048

CsPbI3-cubic

1.02500000000000

6.1349999999999998 0.0000000000000000 0.0000000000000000

0.0000000000000000 6.1349999999999998 0.0000000000000000

0.0000000000000000 0.0000000000000000 6.1349999999999998

Cs Pb I

1 1 3

Direct

0.0000000000000000 0.0000000000000000 0.0000000000000000

0.5000000000000000 0.5000000000000000 0.5000000000000000

0.0000000000000000 0.5000000000000000 0.5000000000000000

0.5000000000000000 0.0000000000000000 0.5000000000000000

0.5000000000000000 0.5000000000000000 0.0000000000000000

FASnI3-cubic

1.035000000000000

6.2201695923162061   -0.0000000282245871   0.0000000000000000

0.0000007823812975   6.0530228143591973   0.0000000000000000

0.0000000000000000   0.0000000000000000   6.1334333839192787

C   N   H   Pb   I

1   2   5   1   3

Direct

0.5000010130040877 0.5286908332526608 0.5000000000000000

0.6860491372913060 0.4306492098538683 0.5000000000000000

0.3139488957057068 0.4306492098453205 0.5000000000000000

0.5000000000030340 0.7104787945879707 0.5000000000000000

0.8202393923822626 0.5293663220288636 0.5000000000000000

0.7071705157137629 0.2620558265487887 0.5000000000000000

0.2928294842809933 0.2620558265453923 0.5000000000000000

0.1797606076200182 0.5293663220208842 0.5000000000000000

0.9999999999987921 0.9497696661704040 0.0000000000000000

0.4999999999983515 0.9640622716905227 0.0000000000000000

0.9999999999986855 0.4466878132019190 0.0000000000000000

0.0000000000029985 0.9246828552534225 0.5000000000000000

$FASnI_3$-orthorhombic

1.035000000000000

8.5658459056244389 0.0338022583601564 -0.0385878766465057

0.0559293510024624 12.0968766655331041 0.0253851807896190

-0.0394434188128824 0.0196393360834740 8.7158064637311110

Pb I C N H

4 12 4 8 20

Direct

0.5079511562182449 0.0005797799328977 0.4963668928576073

0.0155538576678002	0.0007092725349378	0.0034547503551771
0.5066096732666163	0.4998538986955256	0.4973032185935099
0.0128526658943417	0.5013733327859744	0.0018227789997680
0.5113542293145144	0.2512851169795850	0.4904284002785421
0.5059690770703408	0.7511311892462434	0.4881087259278313
0.0243455039350082	0.7510740119262402	0.0061380114365411
0.0288120170319063	0.2509308812014363	0.9969089462369313
0.7814361603747821	0.0009381950567905	0.7309769922389656
0.2176924341209736	0.9992162014942775	0.2918684139757194
0.7292540268309380	0.0112998975694466	0.2185098544134861
0.2827490930947780	0.0036106115106566	0.7703808896903863
0.2140238214000908	0.4959377594706731	0.2936834769705606
0.7825287364763812	0.5004218689801739	0.7278481613978846
0.2842333798993784	0.5075994784683050	0.7735941600065852
0.7244796822905446	0.5086980518810619	0.2159666712553618
0.0015618845088048	0.7095239898679726	0.5020925333506431
0.5151632290557520	0.7279254156190906	0.9910048186091903
0.0034003616205402	0.2090368011988994	0.4963023605736533
0.5110943423121466	0.2269660125447352	0.9874024364038462
0.9180429339470949	0.7612911809510345	0.3982550797521072
0.1014766470065179	0.7545004070009312	0.5960621512416249
0.6184437288726743	0.7518658172496643	0.8876715547977719
0.4441055051558694	0.7997762972713105	0.0779427020794147
0.9231522499750777	0.2584049529224399	0.3878664874321139
0.1055980768338299	0.2556991229372042	0.5862111575958121
0.6238677204569060	0.2516974576981043	0.8944854351956344
0.4401208222164990	0.2967423982828125	0.0774414902538855
0.9851863789898407	0.6198598206042034	0.5128136155793428
0.8427092295539598	0.7165173131488018	0.3310262999001896
0.9246670133243964	0.8448902593007660	0.3776103784997663
0.1269084711267568	0.8375111146452249	0.5974729927821338

0.1620140875601551 0.7039923535819164 0.6716546674729530

0.4851458232197488 0.6402973896850286 0.0054707987415316

0.6694590125852834 0.6885852475646916 0.8261415889057282

0.6530220232218849 0.8316784274403908 0.8628456296706062

0.4683274748515552 0.8827857189656038 0.0752037909033518

0.3636837498560751 0.7743744333836242 0.1565399287828380

0.9814801885500501 0.1206711647951993 0.5149620311442413

0.8453078342819831 0.2128003134851436 0.3242985061239703

0.9359009605368865 0.3404750776081850 0.3597425721462459

0.1354072458227803 0.3377240542971691 0.5785033322894793

0.1629498864461515 0.2078779404470268 0.6671127973568727

0.4719283761854801 0.1406104876417028 0.9886767201922748

0.6747062591178413 0.1896663812866801 0.8303719842532724

0.6641251691549479 0.3312262629103195 0.8784860481495307

0.4731789298150988 0.3779523015035389 0.0873058331243811

0.3476386405692209 0.2716199404818598 0.1421616156357852

$CsSnI_3$-orthorhombic

1.03000000000000

8.6487088099199845 0.0000000000000000 -0.0005590576585399

0.0000000000000000 12.0732946125359923 0.0000000000000000

-0.0004853281656927 0.0000000000000000 8.1898216167006659

Cs I Sn

4 12 4

Direct

0.0728370918164529 0.2500000000000000 0.9751148755566916

0.9274120969952477 0.7500000000000000 0.0248992454268873

0.4275560972050485 0.7500000000000000 0.4750731272257482

0.5728082979335483 0.2500000000000000 0.5249022965552257

0.1982407028883131 0.5329258059963067 0.1925440385115635

0.8019043034626421 0.4670859241900800 0.8075523916553990

0.3019164866063093 0.4670798223126624 0.6923117597735668

0.6982497416491213 0.5327676566524886 0.3075335707549627

0.8019043034626421 0.0329140758099200 0.8075523916553990

0.1982407028883131 0.9670741940036933 0.1925440385115635

0.6982497416491213 0.9672323433475114 0.3075335707549627

0.3019164866063093 0.0329201776873376 0.6923117597735668

0.4985614870107824 0.2500000000000000 0.0643105106349040

0.5014495294247894 0.7500000000000000 0.9359283205763234

0.0014746047071625 0.7500000000000000 0.5644281321087945

0.9990966082302606 0.2500000000000000 0.4354144395121295

0.5000141384432126 0.4999955501547930 0.0000043047820810

0.0000538878510312 0.4999900253457383 0.5000184582240692

0.5000141384432126 0.0000044498452070 0.0000043047820810

0.0000538878510312 0.0000099746542617 0.5000184582240692

CsSnI3-cubic

1.00000000000000

6.2190000000000003 0.0000000000000000 0.0000000000000000

0.0000000000000000 6.2190000000000003 0.0000000000000000

0.0000000000000000 0.0000000000000000 6.2190000000000003

Cs Pb I

1 1 3

Direct

0.0000000000000000 0.0000000000000000 0.0000000000000000

0.5000000000000000 0.5000000000000000 0.5000000000000000

0.0000000000000000 0.5000000000000000 0.5000000000000000

0.5000000000000000 0.0000000000000000 0.5000000000000000

0.5000000000000000 0.5000000000000000 0.0000000000000000

MASnI3-cubic

1.02500000000000

6.0731745396124639   -0.0010743309947183   0.0300803266638338

-0.0010786467260402   6.0629924287505892   0.0002089676266765

0.0304395621010224   0.0002498487131757   6.1330237940473760

C   N   H   Sn   I

1   1   6   1   3

Direct

0.8987365275931865 0.0000134481207965 0.9909359249804979

0.1358550153965581 0.9997785402548942 0.0354665914758385

0.8726758937441303 0.9997515053307495 0.8134723120554028

0.8240698409334186 0.1488681039933297 0.0635942238500746

0.8237021675801657 0.8513901295750443 0.0640011724487266

0.2159546615323151 0.1391089090523536 0.9714449345518190

0.2156096903431433 0.8602234220085876 0.9717698142589697

0.1705972267925233 0.9999862472942453 0.2026158709095540

0.4806462343774882 0.4999609390329525 0.4786849076973851

0.4485867841010034 0.4999303196444984 0.9731206214471015

0.4491222611188945 0.0000280810340598 0.5179706166544307

0.9662527254871733 0.5002313646584824 0.4370639996702081

$MASnI_3$-tetragonal

1.025000000000000

8.4324703751439785 -0.0014233652348497 -0.0005462219600508

0.0051493342194156 8.4418034305723282 0.0007980490596414

0.1085370329072245 0.0822098583503458 12.3771642636398145

C N H Sn I

4 4 24 4 12

Direct

0.0310047763814083 0.4967442625037322 0.2505600656488980

0.5333594200491021 0.0470699605841460 0.2502322783296691

0.0465120456555894 0.5337812256295180 0.7496201763884827

0.4970171865202317 0.0314093780598981 0.7510493721858325

0.9259826960118076 0.6085452125758906 0.1949242829322557

0.4278746098154897 0.9334020434775425 0.1979940161317515

0.9341235768647635	0.4267925345756396	0.6975702219705724
0.6080281578640694	0.9253309511273500	0.6952965355739025
0.6572219593299238	0.0209264807512355	0.2273345459305958
0.5187171583604027	0.0369295383936787	0.3384362002544776
0.5026065307618239	0.1677889312971885	0.2232062062813895
0.4529659050294796	0.8159998891977551	0.2206382430241760
0.4391685271748642	0.9396288706228830	0.1139882105489249
0.3081094647283606	0.9552801932374493	0.2167454795156658
0.9355926759816242	0.5970864788257586	0.1111964361251623
0.9526264120490566	0.7268607189295153	0.2133088727503463
0.8062665943215208	0.5888632654168404	0.2153010095069519
0.1547851126535846	0.5201680150670995	0.2260995024251287
0.9987370782305973	0.3748676831963138	0.2284147444862867
0.0174669682785407	0.5117640763855462	0.3383510751689158
0.9544189015692197	0.3078876451827313	0.7189291747538462
0.9439743924307322	0.4339051023583451	0.6135206255086203
0.8158725494491748	0.4534969774980979	0.7177626560817032
0.1681041397934777	0.5011416063913856	0.7252690517306633
0.0329563159628350	0.5230973816268474	0.8378278313120333
0.0220646687802315	0.6564633721156952	0.7238279627486364
0.7267142894597143	0.9526437561029582	0.7122535204868541
0.5946819683705797	0.9327408944958648	0.6116461279232652
0.5888665761440350	0.8061890629306632	0.7170669900323148
0.5198342882072495	0.1544687455895826	0.7249789191016163
0.5136141066201816	0.0200895235859733	0.8388054519073194
0.3745850558561656	0.9985988239305996	0.7304078268470278
0.5089527856428049	0.5163653881797003	0.9891270880605916
0.5166528083061124	0.5110793488880603	0.4890399879855565
0.0283329705763293	0.0118523621561977	0.4909601333116811
0.0104846587221274	0.0288759166824946	0.9908398646389998
0.7047481228002681	0.8304468606653046	0.9925693187097977

0.3225450108146006 0.2126770639553399 0.9958997780225047

0.2098542513729740 0.7203087350874497 0.9968145339335379

0.8284423656147837 0.3395700749544019 0.9888648828066309

0.3396551477951704 0.8286639232002955 0.4890767479195119

0.7199853397241469 0.2102192291682172 0.4972326941013634

0.8312969442323848 0.7053467417230550 0.4928168833521411

0.2130553677478844 0.3229312741213377 0.4949241378956444

0.5184183268427276 0.5229839970113588 0.2407642345638976

0.5231117898137398 0.5181837569921868 0.7407824583424159

0.0159022310533246 0.0165548305338490 0.7428864818175640

0.0167060452347911 0.0159738810170182 0.2428350099248178

MASnCl3-cubic

1.02500000000000

5.4963258491905451 0.0008873735280529 0.1469738718269263

0.0008399546602409 5.4018715988923960 0.0006809677306016

0.1497648062003817 0.0007048447993364 5.5638535750055951

C N H Sn Cl

1 1 6 1 3

Direct

0.8890755384075462 0.9997217919795034 0.9684461698724363

0.1361112083035323 0.9997067393830221 0.0549990260238076

0.8993397909070140 0.9995136347822040 0.7703556972025112

0.7921043273519146 0.1669917935783118 0.0338680010240253

0.7920639972485617 0.8326403248275867 0.0342853591746106

0.2342580670585477 0.1569490962901483 0.9953832115191261

0.2342690949236399 0.8423935658903758 0.9953765974498694

0.1400921478243617 0.9995611872015360 0.2423348390470466

0.4808368436361334 0.5004199081197243 0.4761663997061092

0.4364930071059732 0.4996559770751290 0.9513545236941354

0.4383244536704325 0.0002831939151875 0.5240661604268055

0.0050129115622966 0.5014652339572194 0.4491517548595496

MASnCl3-orthorhombic

1.02500000000000

7.2362927052732058 0.0000000000000000 0.0000000000000000

0.0000000000000000 10.9610728622243681 0.0000000000000000

0.0000000000000000 0.0000000000000000 8.0981980944705665

C N H Sn Cl

4 4 24 4 12

Direct

0.5352495017594450 0.2500000000000000 0.9224402873250739

0.4647504982405550 0.7500000000000000 0.0775597126749190

0.9647504982405550 0.7500000000000000 0.4224402873250810

0.0352495017594450 0.2500000000000000 0.5775597126749261

0.4639420312109763 0.2500000000000000 0.0921300215030598

0.5360579687890237 0.7500000000000000 0.9078699484969448

0.0360579687890237 0.7500000000000000 0.5921300515030552

0.9639420912109742 0.2500000000000000 0.4078699784969402

0.5111392443440366 0.3271600075785486 0.1558891198375321

0.4888607556559634	0.6728400224214539	0.8441108651624702
0.9888607556559634	0.6728400224214539	0.6558891348375298
0.0111393033440379	0.3271600075785486	0.3441108651624702
0.4888607556559634	0.8271599775785461	0.8441108651624702
0.5111392443440366	0.1728400074214562	0.1558891198375321
0.0111393033440379	0.1728400074214562	0.3441108651624702
0.9888607556559634	0.8271599775785461	0.6558891348375298
0.3191378764291741	0.2500000000000000	0.0916569900283690
0.6808621235708259	0.7500000000000000	0.9083430399716264
0.1808621235708259	0.7500000000000000	0.5916569600283736
0.8191378764291741	0.2500000000000000	0.4083430099716310
0.4852372898530746	0.3322194169958763	0.8582720630511957
0.5147627101469254	0.6677806130041191	0.1417279369488043
0.0147627101469254	0.6677806130041191	0.3582720630511957
0.9852372898530746	0.3322194169958763	0.6417289499488064
0.5147627101469254	0.8322193869958809	0.1417279369488043
0.4852372898530746	0.1677805980041214	0.8582720630511957
0.9852372898530746	0.1677805980041214	0.6417289499488064
0.0147627101469254	0.8322193869958809	0.3582720630511957
0.6875187166861707	0.2500000000000000	0.9260305204597401
0.3124812833138293	0.7500000000000000	0.0739694575402581
0.8124812833138293	0.7500000000000000	0.4260305504597426
0.1875187166861707	0.2500000000000000	0.5739694795402599
0.5000000000000000	0.0000000000000000	0.5000000000000000
0.0000000000000000	0.0000000000000000	0.0000000000000000
0.5000000000000000	0.5000000000000000	0.5000000000000000
0.0000000000000000	0.5000000000000000	0.0000000000000000
0.5381598210221767	0.2500000000000000	0.5078098013613186
0.4618401789778233	0.7500000000000000	0.4921901686386789
0.9618401789778233	0.7500000000000000	0.0078098303613174
0.0381598210221767	0.2500000000000000	0.9921901986386814

0.7927599039183377	0.9737351626425692	0.7147127078373288
0.2072400960816623	0.0262648313574303	0.2852872921626712
0.7072400960816623	0.0262648313574303	0.2147127228373336
0.2927599039183377	0.9737351626425692	0.7852872921626712
0.2072400960816623	0.4737351626425692	0.2852872921626712
0.7927599039183377	0.5262648373574308	0.7147127078373288
0.2927599039183377	0.5262648373574308	0.7852872921626712
0.7072400960816623	0.4737351626425692	0.2147127228373336
